# Patterns of C1-Inhibitor/Plasma Serine Protease Complexes in Healthy Humans and in Hereditary Angioedema Patients

**DOI:** 10.3389/fimmu.2020.00794

**Published:** 2020-05-05

**Authors:** Erika Kajdácsi, Zsófia Jandrasics, Nóra Veszeli, Veronika Makó, Anna Koncz, Dominik Gulyás, Kinga Viktória Köhalmi, György Temesszentandrási, László Cervenak, Péter Gál, József Dobó, Steven de Maat, Coen Maas, Henriette Farkas, Lilian Varga

**Affiliations:** ^1^Research Laboratory, 3rd Department of Internal Medicine, Semmelweis University, Budapest, Hungary; ^2^MTA-SE Research Group of Immunology and Hematology, Hungarian Academy of Sciences and Semmelweis University, Budapest, Hungary; ^3^Hungarian Angioedema Reference Center, 3rd Department of Internal Medicine, Semmelweis University, Budapest, Hungary; ^4^3rd Department of Internal Medicine, Semmelweis University, Budapest, Hungary; ^5^Institute of Enzymology, Research Centre for Natural Sciences, Budapest, Hungary; ^6^Department of Clinical Chemistry and Haematology, University Medical Center Utrecht, Utrecht, Netherlands

**Keywords:** C1-inhibitor, serine protease, kinetic follow-up, activation, hereditary angioedema, HAE attack

## Abstract

C1-inhibitor (C1-INH) is an important regulator of the complement, coagulation, fibrinolytic and contact systems. The quantity of protease/C1-INH complexes in the blood is proportional to the level of the *in vivo* activation of these four cascade-like plasma enzyme systems. Parallel determination of C1-INH-containing activation complexes could be important to understand the regulatory role of C1-INH in diseases such as hereditary angioedema (HAE) due to C1-INH deficiency (C1-INH-HAE). We developed in-house ELISAs to measure the concentration of complexes of C1-INH formed with active proteases: C1r, C1s, MASP-1, MASP-2, plasma kallikrein, factor XIIa, factor XIa, and thrombin, as well as to determine total and functionally active C1-INH. We measured the concentration of the complexes in EDTA plasma from 6 healthy controls, from 5 with type I and 5 with type II C1-INH-HAE patients during symptom-free periods and from five patients during HAE attacks. We also assessed the concentration of these complexes in blood samples taken from one C1-INH-HAE patient during the kinetic follow-up of a HAE attack. The overall pattern of complexed C1-INH was similar in controls and C1-INH-HAE patients. C1-INH formed the highest concentration complexes with C1r and C1s. We observed higher plasma kallikrein/C1-INH complex concentration in both type I and type II C1-INH-HAE, and higher concentration of MASP-1/C1-INH, and MASP-2/C1-INH complexes in type II C1-INH-HAE patients compared to healthy controls and type I patients. Interestingly, none of the C1-INH complex concentrations changed significantly during HAE attacks. During the kinetic follow-up of an HAE attack, the concentration of plasma kallikrein/C1-INH complex was elevated at the onset of the attack. In parallel, C1r, FXIIa and FXIa complexes of C1-INH also tended to be elevated, and the changes in the concentrations of the complexes followed rather rapid kinetics. Our results suggest that the complement classical pathway plays a critical role in the metabolism of C1-INH, however, in C1-INH-HAE, contact system activation is the most significant in this respect. Due to the fast changes in the concentration of complexes, high resolution kinetic follow-up studies are needed to clarify the precise molecular background of C1-INH-HAE pathogenesis.

## Introduction

C1-esterase inhibitor (C1-INH) belongs to the superfamily of serine protease inhibitors. With its mean plasma concentration of 0.25 g/L (3.5 μmol/L) (1), it is among the most abundant protease inhibitors present in the systemic blood circulation. It can inhibit a number of target proteases and, due to its multi-functional role, C1-INH is the key regulator of the complement and contact systems, and is also involved in the control of blood coagulation and fibrinolysis (2). Hereditary or acquired deficiency of C1-INH leads to the occurrence of angioedematous symptoms, affecting subcutaneous or the submucosal tissues (3, 4). Recognizing C1-INH deficiency is indispensable, because, without appropriate treatment, angioedema may severely impair quality of life or even cause death (5). Hereditary angioedema (HAE) resulting from the mutation of the *SERPING1* gene encoding the C1-INH protein (C1-INH-HAE) has two types, both exhibiting autosomal dominant inheritance. In type I, this protein is produced solely by the non-mutated allele, and the concentration of C1-INH in the systemic circulation is usually below half of the normal mean value. In type II C1-INH-HAE, the total concentration of C1-INH is normal or increased, owing to the simultaneous presence of the normal and of a non-functional protein. However, active C1-INH concentration is markedly decreased in both types of C1-INH-HAE (6). The decline of the plasma level of active C1-INH below a critical value (7, 8) is accompanied by the activation of the plasma enzyme systems regulated by this protein. These processes lead to the production of mediators that enhance vascular permeability and, eventually, to angioedema (9). The key mediator is bradykinin (BK), which is released from the high-molecular-weight kininogen during the activation of the kinin system (10), but additional factors can substantially contribute to the edema formation. From these factors, the direct role of active MASP-1, generated during complement activation (11–13), as well as that of thrombin, released upon the activation of the coagulation system (14), have been implicated.

C1-INH has been named after its inhibitory effect on the classical pathway of the complement system; however, it has been found to have a role in the inhibition of a number of additional plasma proteases. C1-INH is the exclusive, natural inhibitor of the active C1r and C1s, and the major inhibitor of active MASP-1 and MASP-2, the components of the complement system (15–18). Furthermore, it is a dominant inhibitor of plasma kallikrein belonging to the contact system (19), as well as of factor XIIa (20) and of coagulation factor XIa (21). C1-INH also inhibits thrombin (22). Moreover, it may have a regulatory role on the fibrinolytic system through the inhibition of plasmin (23), and of tissue-type plasminogen activator (tPA) (24), however, these proteases have a considerable reaction with C1-INH, leading to cleavage of C1-INH rather than their inhibition, which makes the assessment of their complexes unreliable (25–28). We summarized some main properties of these proteases and C1-INH in Table 1. Upon the activation of any of the cascade systems listed above, not only the active protease is inactivated, but also C1-INH is consumed during the inhibitory process. In particular, and typical of serpins, the “suicide” inhibitor C1-INH forms stable, covalent complexes of 1:1 stoichiometry with protease active centers (29, 30). The increased consumption of C1-INH may trigger an HAE attack. In this regard, it is not yet decided which plasma enzyme system(s) is/are responsible for the onset of the HAE attack. The presence of various protease/C1-INH complexes in the plasma reflects the activation of systems regulated by C1-INH. If the concentrations of the protease/C1-INH complexes were known, their relative ratios would give a clue to identifying the activation process in which C1-INH has been consumed. Our goal was to undertake a quantitative comparison of the activation of all C1-INH-regulated pathways, which underlies angioedematous symptoms. Furthermore, we intended to characterize the pathomechanism of the HAE attacks by monitoring the concentrations of protease/C1-INH complexes over time. To ensure comparability, we developed sensitive ELISA assays, where the levels of the protease/C1-INH complexes can be expressed in a standardized fashion, as molar concentrations. Using these new methods, we explored and quantified the differences in the activation of the proteases regulated by C1-INH, which have been found between healthy individuals and C1-INH-HAE patients with or without angioedematous symptoms. In addition, we performed a kinetic follow-up of these changes by monitoring the course of a HAE attack, from the onset until the complete spontaneous resolution of its symptoms.

**TABLE 1:** Summary of some main properties of the studied proteases and C1-INH.

	Plasma concentrations (nM) of the C1-INH and of the zymogen form of its targets (36, 37)	Plasma enzymes system	Other inhibitors
C1-INH_t_	3200	n.a.	n.a.
C1r	581*	Complement-classical pathway	–
C1s	625*	Complement-classical pathway	–
MASP-1	118*	Complement-lectin pathway	Antithrombin, alpha-2-macroglobulin
MASP-2	6.8*	Complement-lectin pathway	Antithrombin
pre-kallikrein	500*	Contact	Alpha-2-macroglobulin
FXII	400*	Contact	Antithrombin, alpha-2-antiplasmin
FXI	31*	Coagulation	Alpha-2-antiplasmin, alpha-1-antitripsin, protein Z dependent protease inhibitor, protease nexin 1, antithrombin, protein C
pro-thrombin	1670*	Coagulation	Antithrombin, heparin cofactor II, tissue factor pathway inhibitor, thrombomodulin

C1-INH_*t*_: total C1-INH concentration. *Mean plasma concentration of the zymogenes from the literature (36, 37).

## Materials and Methods

### Purified Proteins and Antibodies

**FIGURE 1:**
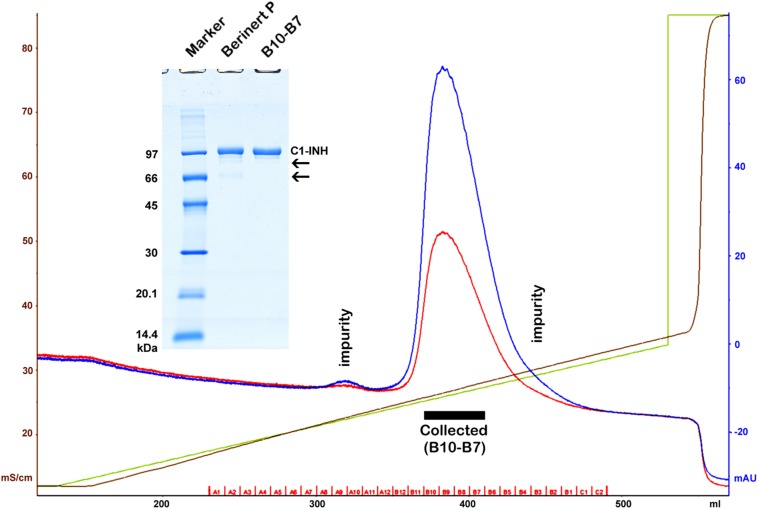
Purification of Berinert P (C1-inhibitor) by anion exchange chromatography. A black rectangle indicates the collected pure fractions representing the middle of the main peak. Blue, red, green and brown lines depict the absorbance at 280 nm, the absorbance at 254 nm, the gradient, and the specific conductivity, respectively. Removed impurities are indicated by labels on the chromatogram, and by arrows on the gel (insertion). SDS-PAGE was performed under reducing conditions. The marker (GE Healthcare Low Molecular Weight marker) is composed of 97, 66, 45, 30, 20.1, and 14.4 kDa proteins.

The active proteases of high purity and the antibodies of the highest specificity – required for the development of sandwich ELISA tests – were obtained from commercial sources. These materials were tested and changed as necessary, and their cross-reactivity was also minimized by altering the reaction conditions. Some reagents were not available; those were prepared by us. Active C1r, C1s, MASP-1, and MASP-2 were produced by recombinant technique. In order to express the measured values as molar concentrations, ultra-pure, active C1-INH had to be obtained. Lyophilized Berinert P was dissolved in water as indicated by the manufacturer. The resulting solution contained glycine, NaCl, and Na-citrate in addition to the protein content. The Berinert P solution was diluted with buffer “A” (100 mM NaCl, 10 mM NaH_2_PO_4_, pH = 7.40) then applied to a Q Sepharose HP (GE Healthcare), 16 mm × 100 mm column, and eluted with a 0–30% “B” gradient (100–370 mM NaCl with 1 M NaCl, 10 mM NaH_2_PO_4_, pH = 7.40) being used as buffer “B.” The chromatogram and the SDS-PAGE analysis are shown in Figure 1. The fractions of the middle of the main peak were pooled. The concentration was determined using the molar extinction coefficient of 27 180 M^–1^ cm^–1^ (31) and the molecular mass of 71 kDa (32) (Figure 1).

The quality of anti-C1-INH IgG obtained from the sera of rabbits immunized with C1-INH was further improved by affinity-purification. In the ELISA test for measuring the concentration of the kallikrein/C1-INH complex, we used 1B12 nanobody reacting with cleaved or complexed C1-INH. We prepared monoclonal anti-C1-INH IgG2a and monoclonal anti-MASP-1 IgG1 (currently not available from commercial sources) in mice to be used in ELISA tests measuring complexes containing MASP-1 and MASP-2. Some of the reagents (recombinant C1s and anti-C1-INH) were biotinylated. The sources and the preparation of the reagents are detailed in Table 2 (33–35).

**TABLE 2:** ELISA protocols for the measurement of concentration of total C1-INH (C1-INH_t_), active C1-INH (C1-INH_a_), and protease/C1-INH complexes.

ELISA	Capture	Plasma sample dilution	Detection antibody	Peroxidase conjugated reagent
C1-INH_t_	Affinity-purified anti-human C1-INH	10000×	Biotinylated anti-human C1-INH	Streptavidin-HRP
C1-INH_a_	Streptavidin	6000×*	Polyclonal anti-human C1-INH	GAR-HRP
C1r/C1-INH	Anti-human C1r	1300×	Biotinylated anti-human-C1-INH	Streptavidin-HRP
C1s/C1-INH	Anti-human C1s	300×	Biotinylated anti-human-C1-INH	Streptavidin-HRP
MASP-1/C1-INH	Anti-human MASP-1	10×	Monoclonal anti-human C1-INH	GAM-HRP
MASP-2/C1-INH	Anti-human MASP-2	10×	Monoclonal anti-human C1-INH	GAM-HRP**
Kallikrein/C1-INH	Anti-human C1-INH	32×	Anti-human kallikrein	RAS-HRP
FXIIa/C1-INH	Anti-human FXII	80×	Affinity purified anti-human C1-INH	GAR-HRP
FXIa/C1-INH	Anti-human FXI	160×	Biotinylated anti-human C1-INH	Streptavidin-HRP
Thrombin/C1-INH	Anti-human thrombin	160×	Affinity purified anti-human C1-INH	GAR-HRP

*Treated with 1.5 nM biotinylated C1s. **Kinetic measurement was done at 530/585 nm using AUR (Amplex Ultrared reagent, Thermo Fisher/Invitrogen) as substrate. Source of used reagents: affinity-purified anti-human C1-INH: rabbit IgG, in-house; biotinylated anti-human C1-INH: rabbit IgG, in-house; polyclonal anti-human C1-INH: rabbit IgG, in-house; streptavidin-HRP: R&D; streptavidin: SIGMA; biotinylated C1s: recombinant in-house; GAR-HRP: goat-anti-rabbit IgG, HRP conjugated, Southern Biotechnology; GAM-HRP: goat-anti-mouse IgG, HRP conjugated, Southern Biotechnology; RAS-HRP: rabbit-anti-sheep IgG, HRP conjugated, Southern Biotechnology; anti-human C1r: goat IgG, R&D; anti-human C1s: goat IgG, Quidel; anti-human MASP-1: monoclonal mouse IgG1, in-house; monoclonal anti-human C1-INH: mouse IgG2a, in-house; anti-human MASP-2: monoclonal rat IgG1, Hycult Biotech; anti-human C1-INH: nanobody, 1B12, in-house (35); anti-human kallikrein: affinity purified sheep IgG, Cederlane; anti-human FXIIa: affinity-purified goat IgG, Cederlane; anti-human FXIa: goat IgG, R&D; anti-human thrombin: sheep IgG, Innovative Research. Detection: TMB substrate (Life Technologies) at 460/620 nm, end-point method, except in the case of MASP-2/C1-INH complex (see above).

### Protease/C1-INH Complexes

The proteases were incubated in molar equivalent ratio with ultra-pure C1-INH for 120 min at 37°C – except for thrombin, which was incubated in the presence of 2 U/ml heparin with eightfold excess of C1-INH to prepare the protease/C1-INH complexes. These complexes were stored for subsequent usage in a 1% BSA-PBS solution, at −80°C. The complexes were checked by reducing SDS-PAGE, and subsequent densitometry was performed to determine what percentage of the known quantity of proteases formed complexes with C1-INH. This ratio was then taken into account to adjust the baseline concentration of the complexes during their use as standards.

### ELISAs

We used 96-well microtiter plates (Nunc^®^ Maxisorp^®^) for every ELISA method; additional details on the material used are set out in Table 2 (33–35). OD was determined with a microplate reader (Infinite^®^ M1000 PRO, Tecan Group Ltd.). Usually – except for kallikrein – the plates were covered with a protease-specific antibody, and the complex content of the samples was determined by adding anti-C1-INH. In every ELISA test, the standards and the plasma samples were measured in duplicates. During complex ELISAs, the protease/C1-INH complexes listed in Tables 2, 3 were used as standards in multiple steps and following linear dilution. Ultra-pure C1-INH was chosen as the standard for sandwich ELISA applied to determine the total C1-INH concentration (C1-INH_t_). To measure the active C1-INH concentration (C1-INH_a_), we adopted the working principle of the commercially available kit by Quidel – that is, the ELISA plate was covered with streptavidin. Purified C1-INH (as standard) or plasma samples were mixed with biotinylated C1s then applied to the streptavidin coated plate. Only those active C1-INH molecules could be detected which had bound to biotinylated C1s. As our ultra-pure C1-INH standard is literally 100-per-cent active protein, the molar concentration of the standard could also be used to express C1-INH activity as molarity.

**TABLE 3:** The specifications of the ELISA tests.

ELISA	Detection limit (pM)	Sensitivity**	Intra-assay CV %	Inter-assay CV%
C1-INH_t_	5.9	1.8 × 10^–6^	6.0	8.0
C1-INH_a_	80	3.5 × 10^–5^	5.7	12.1
C1r/C1-INH	1.5	1.4 × 10^–4^	17.5	17.4
C1s/C1-INH	3.0	5.0 × 10^–4^	11.2	19.3
MASP-1/C1-INH	0.27	2.9 × 10^–4^	8.1	15.5
MASP-2/C1-INH	5.8	8.6 × 10^–2^	16.1	11.1
Kallikrein/C1-INH	8.1	1.6 × 10^–5^	7.9	9.1
FXIIa/C1-INH	2.1	5.5 × 10^–4^	7.5	9.0
FXIa/C1-INH	0.5	7.4 × 10^–3^	11.2	14.9
Thrombin/C1-INH*	1.2	6.0 × 10^–5^	10.9	19.5

C1-INH_*t*_: total C1-INH concentration. C1-INH_*a*_: active C1-INH concentration. *Thrombin was incubated in the presence of 2 U/ml heparin, with an 8-fold excess of C1-INH. **The detection limit of the ELISA (pM) is divided by the plasma concentration of C1-INH_*t*_ or C1-INH_*a*_ or zymogen, respectively (see Table 2, converted into pM). C1r, C1s: recombinant, in-house (33); MASP-1, MASP-2: recombinant, in-house (34); kallikrein, FXIIa, FXIa: Innovative Research; thrombin: Merck.

### Patients and Blood Samples

In a proportion of the experiments, plasma samples anticoagulated with 10 nM EDTA were collected from 6 healthy individuals (5 females and 1 male – aged 28 to 62 years), from 5 patients with type I (4 females and 1 male – aged 21 to 55 years), and additional 5 patients with type II C1-INH-HAE with p.Arg444Cys mutation (4 females and 1 male – aged 42 to 70 years, belonging to 3 families) during symptom-free periods. From 5 of the 10 patients, plasma samples were also obtained during HAE attacks, the properties are detailed in Table 4. The mean (min.-max.) latency between the detection of the HAE symptoms and blood sampling was 4.2 (2–9) h. The mean (min.-max.) interval between symptom-free sampling and sampling during an attack in the same patients was 73 (15–130) days.

**TABLE 4:** The characteristics of the HAE attacks.

C1-INH-HAE type	Localization of the attack	Time between the onset of the attack and the blood sampling (h)	Possible trigger factor of the attack
type II	Abdominal	3	Stress, weather changes
type I	Subcutaneous	9	Physical trauma
type I	Subcutaneous	3	Not known
type I	Abdominal and subcutaneous	2	Not known
type I	Subcutaneous	4	Not known

During the follow-up of the attack, EDTA-plasma samples were collected on 12 occasions, over 96 h of total observation of the course of an HAE attack in a 56-year old female patient with type I C1-INH-HAE. Clinical details of this HAE attack have been described previously by Veszeli et al. (7). Briefly, the patient was symptom-free at the time when the initial and the last blood samples were obtained. During the period in between, erythema marginatum – the only objective prodromal symptom of C1-INH-HAE – developed first, followed by a subcutaneous attack of angioedema involving the left hand and later the right thigh. The patient rated the severity of her symptoms on a visual analog scale (VAS). The blood samples were centrifuged immediately and stored in aliquots at minus 80°C for future use. All laboratory tests were performed on freshly thawed plasma samples. Peripheral blood samples were drawn from all subjects, as prescribed by the study protocol approved by the Government Office of the Capital City Budapest (31110-7/2014/EKU(481/2014), based on the position of the Medical Research Council, after informed consent, in accordance with the Declaration of Helsinki. All participants provided their written informed consent to participate in the study.

### Statistical Analysis and Data Visualization

Results of ELISA measurements were analyzed and interpreted using GraphPad Prism v7.00 (GraphPad Software Inc.) and Microsoft Excel 2010. For statistical analysis, we used one-way ANOVA-multiple comparison.

## Results

### Characterization of the ELISA Tests

We measured the reliability parameters of the ELISA tests, such as detection limits and intra-/inter-assay variation coefficients (expressed as percentages). The detection limits were specified also in relation to the plasma concentrations of the individual proteases (based on literature data: Table 2). The detection limits of the total C1-INH concentration (C1-INH_t_) and the active C1-INH concentration (C1-INH_a_) ELISAs were calculated in relation to the corresponding total C1-INH concentrations we had measured in healthy individuals (Table 2). We provided the details of the analytical parameters of the ELISA tests in Table 3 (36, 37).

### Plasma Levels of C1-INH_t_, C1-INH_a_, and Protease/C1-INH Complexes in Patients and in Controls

Using our set of ELISAs, we determined the C1-INH_t_ and C1-INH_a_ levels in 6 healthy individuals and in C1-INH-HAE patients (5 with type I and 5 with type II HAE) in symptom-free periods. As expected, C1-INH_t_ was markedly lower in samples from patients with type I C1-INH-HAE than in controls, or in patients with type II C1-INH-HAE. In the latter group, C1-INH_t_ concentrations did not differ from those measured in healthy controls. However, C1-INH_a_ was lower in both types of C1-INH-HAE than in healthy individuals. Interestingly, in healthy controls, the mean concentration of C1-INH_a_ was only 64% of the total C1-INH concentration, and a similar ratio was found also in the samples of patients with type I C1-INH-HAE.

We used our ELISAs to measure the plasma concentration of the complexes formed by C1-INH with eight proteases (C1r, C1s, MASP-1, MASP-2, kallikrein, FXIIa, FXIa, and thrombin). Compared with the controls, the concentration of kallikrein/C1-INH complexes was higher both in patients with type I and with type II C1-INH-HAE. The concentration of C1-INH complexed with MASP-1 and MASP-2 were higher only in type II C1-INH-HAE patients compared to the controls. Furthermore, we found that the levels of MASP-1, MASP-2 and FXIa complexes were higher in type II than in type I patients. We did not find significant differences between the patients and the controls or between the two types of patients in the concentration of C1r/C1-INH, C1s/C1-INH, and FXIIa/C1-INH complexes (Table 5).

**TABLE 5:** Concentration of C1-INH_t_, C1-INH_a_ and C1-INH complexes in healthy controls and C1-INH-HAE patients.

	Healthy controls (*n* = 6) [min–max]	Patients with C1-INH-HAE type I (*n* = 5) [min–max]	Patients with C1-INH-HAE type II (*n* = 5) [min–max]
C1-INH_t_ (nM)	3638 [2013–5175]	678.2^∗∗^ [342–1169]	3539^##^ [2673–5957]
C1-INH_a_ (nM)	2201 [1171–3126]	510.9^∗∗∗^ [209.8–994.6]	517.4^∗∗∗^ [315–812.6]
C1r/C1-INH (nM)	28.59 [23.27–34.84]	30.94 [15.58–44.04]	28.54 [20.91–39.97]
C1s/C1-INH (nM)	25.04 [13.88–36.9]	30.46 [16.49–44.59]	35.52 [20.85–44.91]
MASP-1/C1-INH (nM)	0.38 [0.24–0.50]	0.27 [0.20–0.39]	0.55^∗^.^##^ [0.43–0.62]
MASP-2/C1-INH (nM)	0.29 [0.14–0.43]	0.17 [0.07–0.41]	1.27^****^.^####^ [1.00–1.69]
Kallikrein/C1-INH (nM)	0.40 [0.21–0.67]	1.54^∗^ [0.67–2.28]	1.36^∗^ [0.51–2.59]
FXIIa/C1-INH (nM)	0.62 [0.28–1.32]	0.40 [0.29–0.50]	0.65 [0.35–0.94]
FXIa/C1-INH (nM)	1.21 [0.40–1.83]	0.91 [0.63–1.11]	1.79^#^ [1.06–2.32]
Thrombin/C1-INH (nM)	8.47 [3.80–14.75]	8.31 [5.80–9.83]	14.19 [7.30–19.27]

C1-INH_*t*_: total C1-INH concentration. C1-INH_*a*_: active C1-INH concentration. ^∗^Significant difference between healthy controls and patients. #Significant difference between type I and type II patients.^*/#^0.01 < *p* < 0.05. ^**/##^0.001 < *p* < 0.01. ^***/###^0.0001 < *p* < 0.001. ^****/####^*p* < 0.0001. Statistical analysis: ordinary one-way-ANOVA and multiple comparisons.

The concentrations of the C1s, C1r, and thrombin complexed with C1-INH were the highest. The levels of kallikrein and FXIa complexes were lower by an order of magnitude, whereas MASP-1, MASP-2 and FXIIa complexes were the lowest (Table 5).

Finally, we assessed the ratio of the concentrations of protease/C1-INH complexes and the respective plasma zymogens. The latter were adapted from the literature data (36, 37). Interestingly, the proteases could be divided into two characteristic groups: C1r, C1s, MASP-2 and FXIa complexes with C1-INH are significant compared to the zymogen levels (4.9, 4.0, 4.3, and 3.9%, respectively) meanwhile, the ratio of MASP-1, kallikrein, FXIIa, and thrombin complexes with C1-INH to zymogens was rather low (0.32, 0.08, 0.16, and 0.5%, respectively).

### The Ratio of Individual Protease/C1-INH Complexes

**FIGURE 2:**
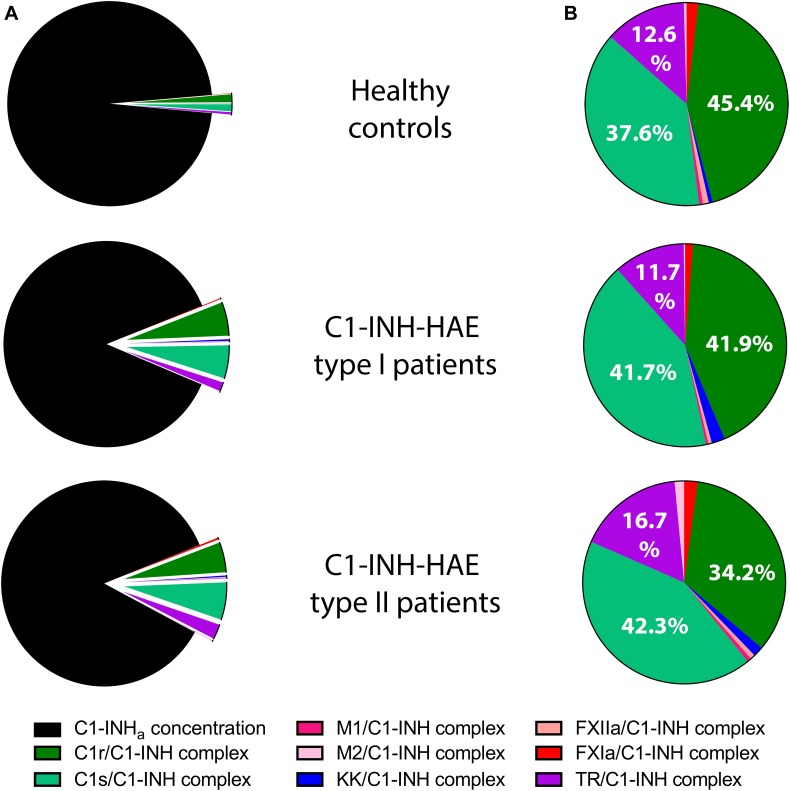
Mean concentration of protease/C1-INH complexes in C1-INH-HAE patients and controls. Comparison of the concentration of protease/C1-INH complexes relative to active C1-INH concentration (C1-INH_a_) **(A)**, and pattern of individual protease/C1-INH complexes **(B)**. M1 = MASP-1, M2 = MASP-2, KK = Kallikrein, and TR = Thrombin.

The C1-INH_a_ level was much lower in the patients, therefore the protease/C1-INH to C1-INH_a_ ratio was higher in patients: 14% in Type I, and 16% in type II patients compared to controls (3%) (Figure 2A). However, summed concentrations of the individual protease/C1-INH complexes were similar in healthy controls (65 nM), in type I (73 nM), and in type II (84 nM) C1-INH-HAE patients. Moreover, the ratio of C1-INH complexes compared to each other was also similar in C1-INH-HAE patients and in controls (Figure 2B).

### Plasma Levels of C1-INH_t_, C1-INH_a_, and Protease/C1-INH Complexes in Patients During HAE Attacks

**FIGURE 3:**
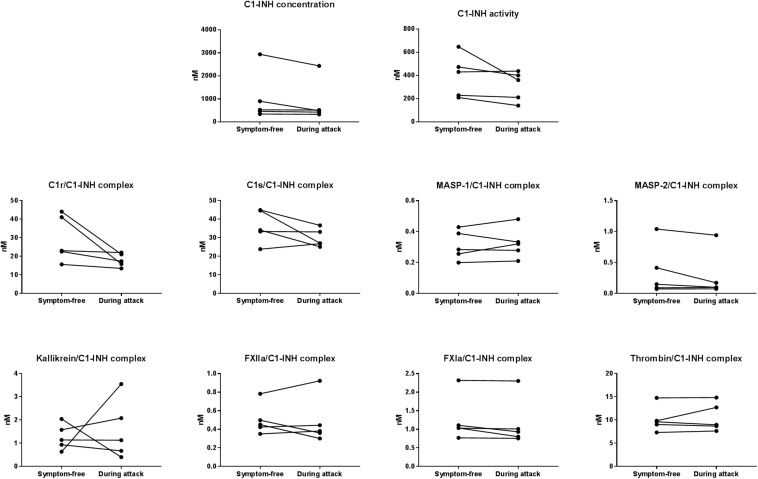
Mean concentration of protease/C1-INH complexes in C1-INH-HAE patients during attack and in symptom-free period. Comparison of total C1-INH concentration (C1-INH_t_), active C1-INH concentration (C1-INH_a_); and concentrations of C1r-, C1s-, MASP- 1-, MASP- 2-, kallikrein-, FXIIa-, FXIa-, and thrombin/C1-INH complexes in patients with C1-INH-HAE in symptom-free periods and during HAE attacks.

In five patients (4 with type I and 1 with type II C1-INH-HAE), samples obtained during HAE attacks were also available. The mean time elapsed between the onset of the attacks and the blood sampling was 4.2 h. The clinical properties of the HAE attacks are shown in Table 4. None of the C1-INH_t_, C1-INH_a_, or C1-INH complexed with the proteases showed any marked changes in the samples obtained during an HAE attack, compared to symptom-free periods (Figure 3).

### The Kinetic Follow-up of the C1-INH_t_, C1-INH_a_, and the Levels of the Protease/C1-INH Complexes During an HAE Attack

**FIGURE 4:**
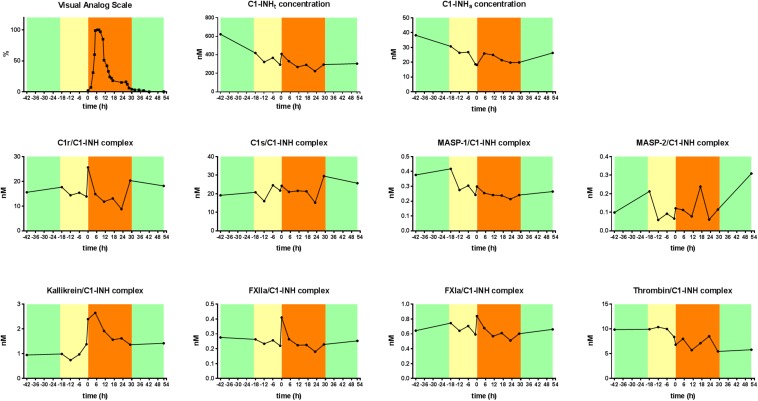
Kinetic follow-up of protease/C1-INH complexes during an angioedematous attack. Kinetics of total C1-INH concentration (C1-INH_t_), active C1-INH concentration (C1-INH_a_) and concentrations of C1r-, C1s-, MASP- 1-, MASP- 2-, kallikrein-, FXIIa-, FXIa-, and thrombin/C1-INH, complexes during an HAE attack of a type I C1-INH-HAE patient. Severity of angioedematous symptoms were assessed by the patient in a visual analog scale (VAS, 0–100%). Symptom-free periods are marked with green, prodromal phase with yellow and HAE attack phase with orange colors.

The levels of C1-INH_t_, and C1-INH_a_, as well as those of the C1-INH complexed with the proteases were measured in blood samples obtained during a subcutaneous attack experienced by a female patient with type I C1-INH-HAE. C1-INH_a_ level was almost stable during the prodromal phase, however, it decreased right before the onset of the HAE attack. The concentration of the kallikrein/C1-INH complex increased significantly at the onset of the HAE attack and remained high for 6 h, then it started to decrease gradually. The kallikrein/C1-INH complex concentrations correlated well with the severity score of angioedematous symptoms. The concentrations of the C1r/C1-INH, FXIIa/C1-INH, and FXIa/C1-INH complexes also reached a distinct peak at the onset of the HAE attack however, their concentration changes were not as remarkable as those of kallikrein complex (Figure 4).

## Discussion

We have applied and developed assays for the simultaneous determination of the molar concentrations of total C1-INH, active C1-INH and C1-INH complexed with eight different plasma serine proteases including the first assay to measure C1r/C1-INH complex. This made it possible to monitor the distribution of C1-INH among plasma enzyme systems, and to calculate their proportion normalized to total or active C1-INH concentration. By now, sensitive ELISA or RIA methods have been developed for the detection of nearly all target proteases (C1s, MASP-1, MASP-2, kallikrein, FXIIa, FXIa, and thrombin) that form complexes with C1-INH (21, 38–45). However, only few of the studies outlined above determined the concentration values in moles per liter, and not as relative units (21, 40–42). Furthermore, in the previous studies, only maximum three different complexes were measured simultaneously (41, 46, 47). Using our C1-INH complex ELISA set, we successfully measured the concentration of total C1-INH, active C1-INH, and complexed C1-INH in six healthy controls, 5–5 C1-INH-HAE type I and type II patients in symptom-free periods, five patients during HAE attacks, as well as during a kinetic follow-up study of an HAE attack in one C1-INH-HAE patient from the beginning until the spontaneous termination of HAE symptoms.

Previous studies found differences between healthy controls and C1-INH-HAE patients only in the level of C1s/C1-INH and MASP-1/C1-INH complexes. Higher C1s/C1-INH (41, 48, 49) and lower MASP-1/C1-INH complex levels (44) were detected in patients compared to controls. We could not confirm these findings, as the concentration and the pattern of C1-INH complexed with the different proteases were very similar in the patients and in healthy individuals. We are aware that the sample collection and handling certainly differs between this study and the previous studies, furthermore, the different methods and the low sample size may also influence the outcome of the study. Compared with active C1-INH concentration present in the systemic circulation, the summed concentration of all protease/C1-INH complexes represents only a minor fraction. This could lead to the conclusion that low active C1-INH concentration might be of sufficient capacity to inhibit target proteases, however, even in C1-INH-HAE patients, only a fraction of the active C1-INH concentration consumed for the production of complexes was detectable in the circulation. This is in concert with the results previously published by Cugno et al. (41) where they determined the concentration of kallikrein/C1-INH complex and the concentration of the total C1-INH as well. The ratio of kallikrein/C1-INH complex to C1-INH_t_ was 0.00009 in their study. We found practically the same ratio (Table 5; 0.40 nM/3638 nM = 0.0001). Surprisingly, we found that in healthy controls, the concentration of C1-INH_a_ was just 64% of that of C1-INH_t_. Although we have detected several low density bands with molecular weight lower than 105 kD in Western-blot developed by anti-C1-INH antibodies, these together cannot account for the gap between the active-plus complexed- and the total concentration of C1-INH. Therefore, we cannot exclude that our ELISA overestimates C1-INH_t_ compared to C1-INH_a_.

According to our results, C1-INH forms complexes predominantly with C1r and C1s. This is also in agreement with the previously published data by Cugno et al., where they found more than ten-fold higher concentration of C1s/C1-INH complex than that of kallikrein/C1-INH complex (41). One or more of the followings can explain this finding: (1) C1-INH is the exclusive inhibitor of these factors, (2) the concentration of these enzymes is among the highest of serine proteases in the circulation, (3) these complexes may have the lowest clearance speed, and (4) the basal activation rate of these proteases may be the highest among the target enzymes of C1-INH.

Furthermore, the relatively high ratio (∼4%) of C1r-, C1s-, and MASP-2/C1-INH complexes compared to the respective zymogens does not exclude that not just the complement alternative pathway has a tick-over mechanism, but the classical and the lectin pathways have a similar fluid-phase basal-level activation as well. It is also true for FXI, a key component of the coagulation pathway.

Meanwhile, we measured significantly higher concentrations of kallikrein/C1-INH complexes in C1-INH-HAE patients, and a higher concentration of MASP-1 and MASP-2 complexes were observed in type II patients compared to healthy controls. These results may suggest the prominent role of the complement lectin pathway – besides contact system activation – in the pathomechanism of C1-INH-HAE. Furthermore, we found that, in type II patients, the concentration of MASP-1, MASP-2, and FXIa/C1-INH complexes were significantly higher than in type I patients, which raises the unexpected question of whether there is some difference in the molecular pathomechanism of the type II C1-INH-HAE with p.Arg444Cys mutation. Nevertheless, we have to be careful when interpreting these results, since the 5 type II C1-INH-HAE patients belonged to three families, which may drift our data.

Previously, the level of protease/C1-INH complexes were scarcely studied during HAE attacks (50, 51) and only in one study was found difference between the level of C1s/C1-INH complexes in HAE attacks and symptom-free periods (50). However, the samples with elevated complexes during HAE attacks were not obtained from the same patients from which the symptom-free samples were obtained. We measured the concentrations in the during attack and symptom-free samples of the same five patients and did not find any difference in total C1-INH, active C1-INH, and complexed C1-INH concentrations between the symptom-free and during attack state of the patients as Nielsen et al. suggested (51). Our hypothesis, i.e., the HAE attack phase would differ from the symptom-free phase in terms of C1-INH (total, active or complexed) concentration, was built upon the fact that C1-INH plays a central role in the pathomechanism of C1-INH-HAE. However, our results may not contradict the initial hypothesis and the previous knowledge about the pathogenesis, since the rapid kinetics of the complexes measured in the follow-up part of the study suggested that differences could only be detected soon after the onset of the HAE attacks. Nevertheless, involving more patients may reveal minor differences between symptom-free and during attack phases in C1-INH-HAE.

As shown by the detailed, kinetic follow-up of an HAE attack, C1-INH_a_ concentration decreased exactly before the onset of the HAE attack, which supports that C1-INH may have a direct regulating effect on the initiation of the HAE attacks. Some cascade systems controlled by C1-INH undergo intense activation, i.e., at the onset of angioedematous symptoms, the concentrations of kallikrein/C1-INH complex were elevated and exhibited a further increase in a good correlation with the VAS score. In parallel with the onset of the symptoms, C1-INH complexed with C1r, FXIIa, and FXIa also tended to increase rapidly, but returned to baseline within 6 h, which may suggest a very fast clearance rate of these complexes. Our results suggest that, besides the contact system, complement system activation may also be involved in the initiation of the HAE attacks. It is further supported that we found significant elevation in C4a concentration during the prodromal phase (7). Nevertheless, to resolve the molecular basis of HAE attacks, an even more frequent blood sampling and more detailed analysis would be required. Moreover, we have very scarce information on the clearance mechanisms and kinetics of the various protease/C1-INH complexes (52), which underlines that, besides the systemic (i.e., plasma) investigations, the assessment of local events during attacks would be necessary.

Our goal was to measure the interactions between C1-INH and its target proteases in a comparable manner. In this respect, ours is a pioneering study, since no similar comprehensive characterization has ever been made. It is also unique in that it has explored the kinetics of the regulation of serine proteases by C1-INH in a single clinical case. Based on our experiments, we can conclude that the pattern of protease/C1-INH complexes are quite similar in healthy controls and in C1-INH-HAE patients, and that C1-INH metabolism depends mainly on the contact- and complement system. In addition to C1-INH-HAE, studying the activation of the plasma enzyme systems controlled by C1-INH is also important in other conditions (e.g., sepsis, cardiopulmonary bypass, SLE, and polytrauma), and has already been undertaken, although by monitoring fewer parameters (40, 44, 45, 53). The method developed by us might allow for a more complex exploration of the pathomechanism of these disorders.

## Data Availability Statement

The datasets generated for this study are available on request to the corresponding author.

## Ethics Statement

Peripheral blood samples were drawn from all subjects, as prescribed by the study protocol approved by the Government Office of the Capital City Budapest (31110-7/2014/EKU (481/2014), based on the position of the Medical Research Council, after informed consent, in accordance with the Declaration of Helsinki. All participants provided their written informed consent to participate in the study.

## Author Contributions

LV designed and supervised the study, and wrote the original manuscript. EK coordinated the development of the ELISAs, participated in the blood sampling, carried out the purification of polyclonal antibody against C1-INH, carried out the measurement of C1-INH complexes, carried out the statistical analyses and data display, and wrote the original manuscript. NV participated in the blood sampling, carried out of purification of polyclonal antibody against C1-INH, took part in developing of the ELISAs, carried out the measurement of C1-INH complexes AK and DG took part in developing the ELISAs. VM developed monoclonal antibodies against MASP-1 and C1-INH, and carried out the measurement of C1-INH complexes. ZJ carried out the measurement of C1-INH complexes. LC took part in developing the ELISAs, and carried out the measurement of C1-INH complexes. PG and JD produced recombinant C1s, C1r, MASP-1, and MASP-2, carried out the affinity purification the polyclonal antibody against C1-INH, carried out the ultra purification of Berinert P (C1-INH). CM and SM developed the nanobody against complexed C1-INH, and developed the original protocol of kallikrein/C1-INH ELISA. KK and GT provided the medical background during blood sampling. HF is the head of the Hungarian Angioedema Reference Center, and took care of the HAE patients and provided the clinical data. All authors contributed to the drafting or revision of the manuscript and provided their final approval of the manuscript for submission.

## Conflict of Interest

The authors declare that the research was conducted in the absence of any commercial or financial relationships that could be construed as a potential conflict of interest.
